# Biogenic Synthesis of CuO, ZnO, and CuO–ZnO Nanoparticles Using Leaf Extracts of *Dovyalis caffra* and Their Biological Properties

**DOI:** 10.3390/molecules27103206

**Published:** 2022-05-17

**Authors:** Jerry O. Adeyemi, Damian C. Onwudiwe, Adebola O. Oyedeji

**Affiliations:** 1 Department of Chemical and Physical Sciences, Faculty of Natural Sciences, Walter Sisulu University, Mthatha 5099, South Africa; aoyedeji@wsu.ac.za; 2 Department of Chemistry, Faculty of Natural and Agricultural Science, North-West University, Private Bag X2046, Mmabatho 2735, South Africa; damian.onwudiwe@nwu.ac.za; 3 Material Science Innovation and Modelling (MaSIM) Research Focus Area, Faculty of Natural and Agricultural Science, North-West University, Mafikeng Campus, Private Bag X2046, Mmabatho 2735, South Africa

**Keywords:** *Dovyalis caffra*, zincite, tenorite, nanocomposite, antioxidant, anticancer

## Abstract

Biogenic metal oxide nanoparticles (NPs) have emerged as a useful tool in biology due to their biocompatibility properties with most biological systems. In this study, we report the synthesis of copper oxide (CuO), zinc oxide (ZnO) nanoparticles (NPs), and their nanocomposite (CuO–ZnO) prepared using the phytochemical extracts from the leaves of *Dovyalis caffra* (kei apple). The physicochemical properties of these nanomaterials were established using some characterization techniques including X-ray diffraction analysis (XRD), ultraviolet-visible spectroscopy (UV-vis), scanning electron microscopy (SEM), transmission electron microscopy (TEM), and energy-dispersive X-ray spectroscopy (EDX). The XRD result confirmed the presence of a monoclinic CuO (Tenorite), and a hexagonal ZnO (Zincite) nanoparticles phase, which were both confirmed in the CuO–ZnO composite. The electron microscopy of the CuO–ZnO, CuO, and ZnO NPs showed a mixture of nano-scale sizes and spherical/short-rod morphologies, with some agglomeration. In the constituent’s analysis (EDX), no unwanted peak was found, which showed the absence of impurities. Antioxidant properties of the nanoparticles was studied, which confirmed that CuO–ZnO nanocomposite exhibited better scavenging potential than the individual metal oxide nanoparticles (CuO, and ZnO), and ascorbic acid with respect to their minimum inhibitory concentration (IC_50_) values. Similarly, the in vitro anticancer studies using MCF7 breast cancer cell lines indicated a concentration-dependent profile with the CuO–ZnO nanocomposite having the best activity over the respective metal oxides, but slightly lower than the standard 5-Fluorouracil drug.

## 1. Introduction

Cancer has continued to be one of the leading causes of death all over the world and in 2020, was reported to account for about 10 million deaths, worldwide [1]. It has been found to manifest because of the disruption of cell growth by some genetic pre-dispositions (oncogenes and genes responsible for DNA repair, apoptosis, and tumor suppression) and some environmental factors such as ionizing radiation and exposure to dangerous chemicals including tobacco smokes [2]. Morbidity projection into the year 2030 has predicted that about 26 million deaths could be recorded if this menace is not curbed [3]. Therefore, a lot of efforts including immunotherapy, targeted therapy, photodynamic stem cell transplant, chemotherapy, radiotherapy, and hyperthermia have all been employed over the years. However, this problem persists despite these tremendous efforts and resources invested in finding a cure [4]. Several challenges have emerged as the reason for the continuous persistence but most notably are the metastatic nature of cancer, the viability of the stem cells, the discovery of numerous onco-types, and the drug specificity to different cell lines [5]. Several conventional chemotherapeutic treatments have been used over the years, these treatment methods are, nevertheless, plagued with diverse side effects emanating from their inability to effectively penetrate solid tumors (thereby failing to kill the cancerous cells) and their non-selectivity towards the desired cell lines [6]. The emerging side effects include alopecia (hair loss), myelosuppression, thrombocytopenia, mucositis, and organ dysfunction; these side effects may lead to delay in treatment, dose reduction, or discontinuance of the given drugs [6]. Different methods have thus been used to circumvent these side effects in recent years, which has resulted in the discovery of some biogenetic agents such as Madagascar Periwinkle (*Catharanthus roseus*), which has given a new insight into the role of nature, especially medicinal plants in cancer therapy. The extracts of these plant materials were found to possess cytotoxic properties which are cancer cell-specific with little or no side effects unlike their synthetic counterparts [7,8].

It is well documented that over 60% of most currently used medicines are derived either directly or indirectly from a natural source such as animals, plants, and minerals [9]. This has brought about the increased interest in using these biogenetic materials for the synthesis of nanoparticles which will be used for biological applications due to their propensity for increased biocompatibility with biological cells. Therefore, several metal-based nanoparticles designed to function in biological cells were prepared from diverse arrays of plant materials, including stems, leaves, fruits, and roots [10,11,12,13]. These nanoparticles do not only offer medicinal benefits but were reported to possess multiple oxidation states and large surface areas, thereby allowing for high reactivity [14]. These metal-based nanoparticles prepared using the biomimetic routes offer better chemotherapeutic advantages than the conventional ones by improved efficacy, increasing half-life, and slowly releasing drugs into the biological system [15]. A notable group amongst the metal-based nanoparticles is the metal oxide nanoparticles.

Metal oxide nanoparticles have received considerable attention in the past few decades owing to their useful properties and applications in many areas of life. Amongst these are copper oxide (CuO) [16] and zinc oxide (ZnO) nanoparticle [17,18,19]. These two oxides are among the benign oxides that have shown much diverse usefulness as biological agents. Their diverse application and the recent increased attention given to them are related to their unique properties, which are associated with their size, shapes, and compositions [20]. CuO has high heat of conductivity, electron correlation effects, and spin dynamics [21]. These properties have led to a wide range of applications in drug delivery [22], bioimaging [23], photocatalysis [24,25], and antioxidant agents [26]. It was also used as an antimicrobial agent against bacterial and fungi [20]. Similarly, over the years, ZnO NPs have become very useful due to their green properties, excellent stability, low price, and different facile preparation routes [17,18,19]. They were established to possess useful antimicrobial properties [27]. Zinc oxide nanoparticles have been reported to inhibit the growth of microorganisms including bacteria and fungi. Their mechanism of action was suggested to involve activation of the ZnO NPs by light penetrating the cell wall of the organism through diffusion. This phenomenon then helps the rupturing of the cell wall, thereby leading to the accumulation of these material in the cytoplasm [27], which then results in cell apoptosis due to the interaction with other biomolecules found in the cytoplasm [27]. Zinc oxide NPs were also reported to inhibit the activation of NF-kB by reducing the mRNA expression of inflamed cytokines [27]. Furthermore, they have become important ingredients in our day-to-day products such as sunscreen, ointment for pain, enzymes, and itch relief agents. Due to their wide bandgap, they are also good light absorbers with great efficiency [27]. It is, therefore, evident that the biological importance of CuO and ZnO can never be over emphasized. However, the effectiveness as biological agents has been found to be dependent on the particles’ size, morphology, concentration, exposure time, biocompatibility, and pH [27].

Generally, the use of nanomaterials is dependent on properties such as morphology, size, and homogeneity. These properties are dependent on several other factors including the method of synthesis and precursor type. Several methods were used for the preparation of metal-based nanoparticles, such as sol–gel, thermal decomposition of a precursor compound, microwave, and chemical precipitation [28,29,30]. However, due to the environmental challenges associated with the use of these chemical methods and the increased desire for more environmentally friendly routes to materials synthesis, photochemical synthesis of nanomaterials has emerged as a famous route to nanoparticles’ synthesis [31]. This approach takes advantage of the presence of different phytochemicals such as flavonoids, polyphenol compounds, and ascorbic acids in plant materials [26]. Thus, metal oxide nanoparticles were prepared using the extracts of plants such as *Gundelia tournefortii* [31], *Juglans regia* [26], *Abutilon indicum* [16], Aloe, and *barbadensis Miller* [28]. Despite the extensive literature and years of research that exist on the use of plant materials for the synthesis, no report exists for the synthesis of NPs using Kei apple (*Dovyalis caffra*) leaves. The synthesis of multimetallic and bimetallic NPs has continued to gain attention in recent times. However, their synthesis is time-consuming, costly, and not void of hazardous toxic substances. Hence, they were generated using different eco-friendly methods such as those prepared using different plant materials. A prominent example is the CuO–ZnO nanoparticles due to their ability to individually confer different biological actions synergistically and their biocompatibility with human cells [32]. Several eco-friendly methods using plants such as *Cardiospermum halicacabum* and *Mangifera indica* leaves extracts were used to prepare ZnO nanoparticles that have shown useful antitumor properties against A375 and A549 cell lines. Similarly, CuO nanoparticles from a green synthetic route were also reported to inhibit the continuous proliferation of A549 cancer cells by stimulating apoptosis [33]. Furthermore, ZnO was reported to inhibit or prevent DNA damage by aiding the production of reactive oxygen species (ROS) due to their unique electrostatic behavior. A recent study has reported biogenetic ZnO NPs that showed high potential to induce apoptosis in A375 cancer cells by increasing the amount of ROS [34]. It was reported that ZnO, at physiological pH, increases the phagocytic activity of the body and in turn brings about the death of the cancerous cells [32]. Additionally, CuO NPs have induced cell apoptosis by increasing the phagocytic activity of the body at certain physiological pH [32].

In this study, therefore, we report the use of Kei apple leaves (*Dovyalis caffra*) for the synthesis of nanoparticles. To the best of our knowledge, the synthesis of CuO, ZnO NPs, and CuO–ZnO nanocomposites are yet to be mediated using extracts form this plant’s leaves. The antioxidant and cytotoxicity potentials of these nanomaterials were then examined using the 1,1-diphenyl-2-picryhydrazyl (DPPH) assay and MTT assay method, respectively.

## 2. Results

### 2.1. Synthesis

**Figure 1:**
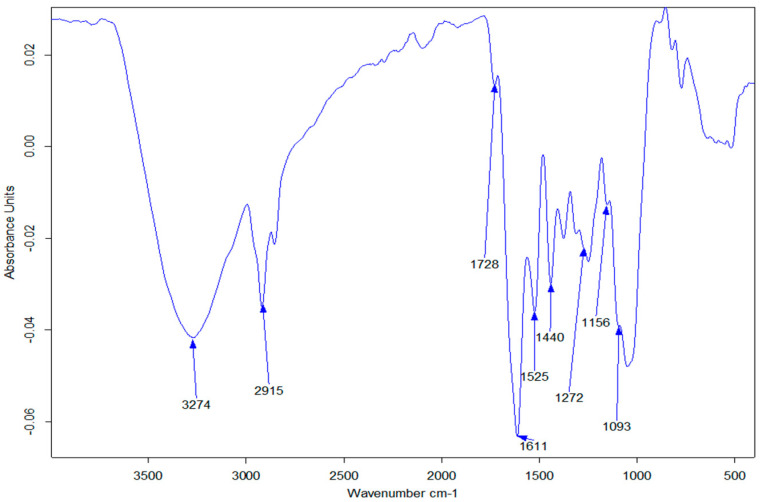
FT-IR spectrum of *Dovyalis caffra* leaf extracts.

**Figure 2:**
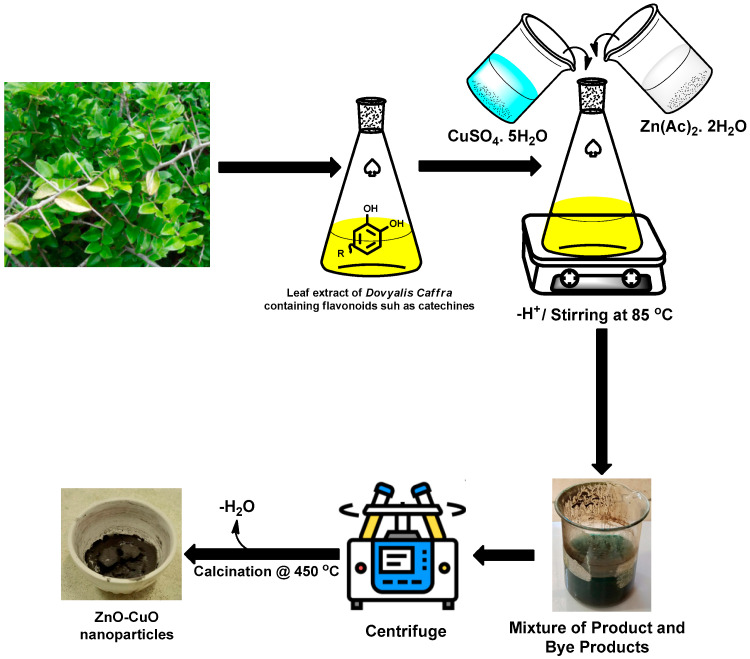
Schematic representation for the preparation of CuO–ZnO using *Dovyalis caffra* extracts.

Plants containing high content of polyphenolic compounds have already shown the capacity to adequately reduce different metal salts such as silver into their metallic nanomaterials due to the reducing potentials of these phenolic compounds [35,36]. As already established by previous studies, all parts of the *Dovyalis caffra* plant have a high content of polyphenolic compounds and flavonoids, which could mediate the formation of metal oxide NPs [37]. Hence, several classes of phytochemicals including flavonoids and total phenolics were identified and quantified for *Dovyalis caffra* in the literature with varying concertation due to factors such as soil type, season, and climate [37]. Thus, based on these studies, 30 phenolic compounds have been quantified by different reports [37]. In this current study, therefore, the identification of the phytochemical constituents on the leaves was carried out using FTIR spectroscopy (see Figure 1), which showed that the plant extracts possess compounds bearing the -OH, -CO, -C=H, and -C-H functional group due to the appearance of peaks at around 3274(broad), 1728, 1440 and 1093 cm^−1^. The appearance of the broad -OH peak suggests the presence of phenolic compounds, which is possibly responsible for the stabilization process of the nanoparticles. The appearance of the carbonyl indicates the possible presence of ketones, esters, and aldehydes [38,39]. The probable mechanism involved in this synthetic process is presented in Figure 2, similar to other metal oxides [40]. The formation process, as shown in Figure 1, is initiated via the ionization of the metal salt solutions to give the Cu^2+^ and SO_4_^2−^ ions in the solution. This step was followed by the formation of copper and zinc complexes due to the hydroxyl group from the polyphenolic compounds. Other phytochemicals such as catechins, which have already been reported to be present in the plant, bind to the metal ion and consequently trigger (stabilizes) the formation of respective hydroxides [40]. The product was collected and centrifuged and upon calcination of the resulting hydroxides, water molecules were removed resulting in the formation of the metal oxide NPs product. The synthesis of ZnO and CuO and their respective bimetallic nanocomposites followed this mechanism of synthesis.

### 2.2. Phase Identification and Structural Properties of the Prepared CuO

**Figure 3:**
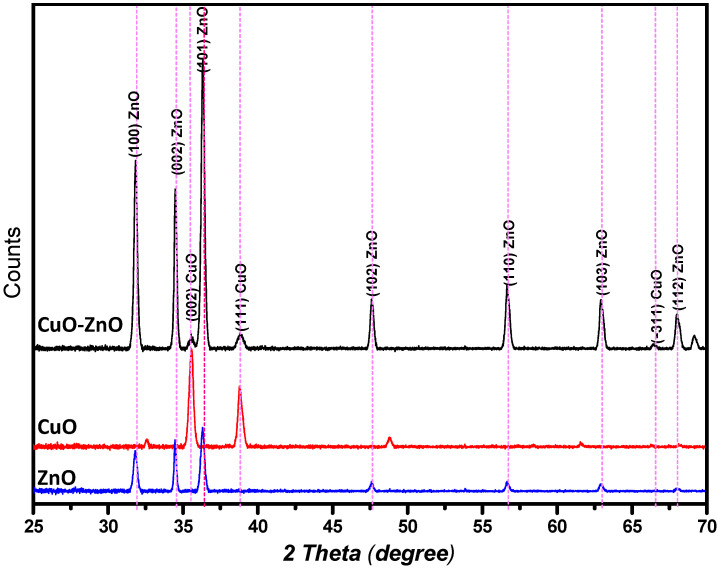
XRD patterns of the prepared nanoparticles from the aqueous extract of Kei apple leaves.

The phase identification and the crystallinity of the obtained CuO, ZnO, and CuO–ZnO nanoparticles were confirmed using the XRD analysis, and the obtained diffractograms are presented in Figure 3. From the diffraction pattern of the CuO–ZnO composite, the peaks identified at 2θ = 35.41, 38.70, and 66.22° were ascribed to the lattice indices (0 0 2), (1 1 1), and (−3 1 1), respectively, which is consistent with the monoclinic phase of the standard JCPDS no 00-048-1548 for CuO (Tenorite) nanoparticles [41]. Similarly, the identified peaks at 2θ = 31.77, 34.42, 36.25, 47.53, 56.60, 62.86, and 67.96° are ascribed to the (1 0 0), (0 0 2), (1 0 1), (1 0 2), (1 1 0), (1 0 3), and (1 1 2), lattice indices respectively. This is also consistent with the hexagonal phase of the standard JCPDS no 00-036-1451 for ZnO (Zincite) nanoparticles [42]. The diffraction peaks of CuO–ZnO nanocomposite thus reflect substantially, adequate integration of both metal oxide nanoparticles, similar to previous report in which composites with <15% Cu shows one-phase wurtzite-like Cu_x_Zn_1−x_O Form [43]. Comparing the obtained diffraction peaks of both CuO and ZnO nanoparticles with the synthesized CuO–ZnO nanocomposites, there is an increase in the intensity of the peaks which is indicative of high crystallinity. The (1 0 1) peak has the most intensity which suggests that most of the crystals are dominated by the (1 0 1) facets. Furthermore, as seen in Figure 2, the intensities of the peaks associated with the ZnO in the CuO–ZnO diffraction pattern seemed higher than those of CuO NPs. This is suggestive of a larger percentage of the bimetallic structure of zinc–copper NPs and high crystallinity [32]. However, the lower crystallization and intensity of the CuO NPs peaks in the composites have been associated with the coating role of ZnO NPs on the formed CuO NPs, which consequently protects the copper layer from further oxidation [44]. The CuO and ZnO nanoparticles, which are often made from copper sulfate and zinc acetate precursor salts, were reported to show good purity [30,45]. Hence, no impurity was found in the nanoparticles and their composite. Furthermore, from the diffraction patterns of the nanocomposite, the crystallite size, D, was calculated using Scherrer’s equation (Equation (1)).

(1) $$D=\frac{0.9\lambda }{FWHM\cdot \;Cos\;\theta }$$

where λ is the copper Kα wavelength at 1.5418 Å. The full width at half maximum (*FWHM*) of the peak with the highest intensity (2θ = 35.6°) and the angle parameter (*θ*) was estimated using the OriginPro 8. The obtained particle sizes found for the CuO–ZnO, CuO, and ZnO nanoparticles were 23.21, 29.19, and 25.29 nm, respectively. The sizes and the pattern of trend (that is, CuO–ZnO > CuO > ZnO) observed for these three materials seemed to be in good agreement with those found in earlier study reported by Mohammadi et al. using *Mentha longifolia* leaf extract [17].

### 2.3. Surface Morphology and Elemental Analysis of CuO Nanoflakes

**Figure 4:**
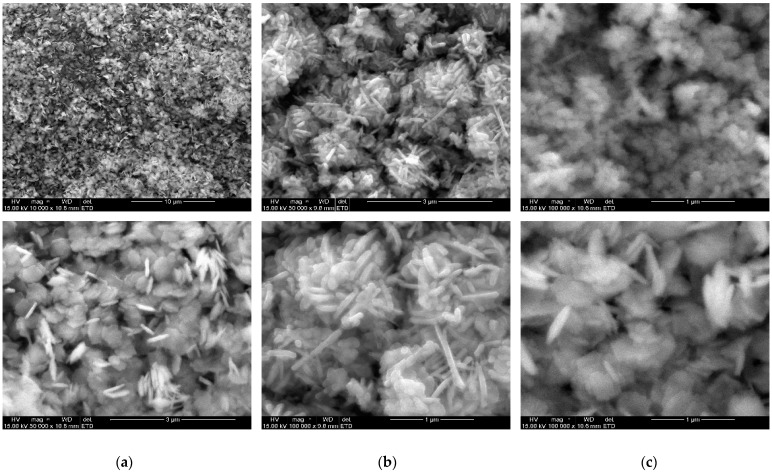
SEM micrograph images reveal the morphological features of the prepared (**a**) CuO (**b**) ZnO (**c**) CuO–ZnO showing nanoparticles at different magnifications.

**Figure 5:**
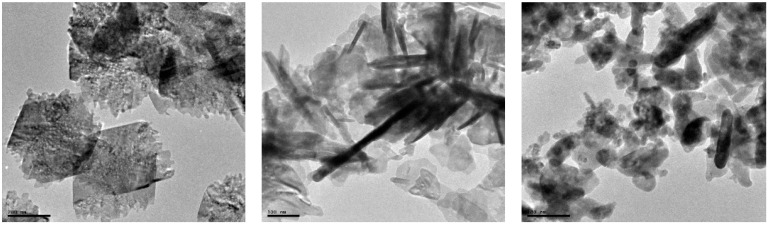

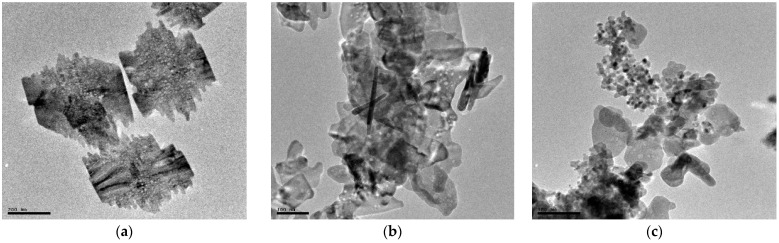
TEM micrograph images reveal the morphological features of the prepared (**a**) CuO (**b**) ZnO (**c**) CuO–ZnO showing nanoparticles at different magnifications.

The morphological properties (size, shape, and roughness) of the nanoparticles were reported to influence their applications, which makes the microscopic investigation a useful technique in establishing the nature of a nanomaterial [43]. The morphological properties of the nanoparticles and their composite were studied using scanning electron and transmission electron microscope (SEM and TEM), at high and low magnifications. Figure 4a–c presents the SEM micrographs, while Figure 5a–c are representative TEM micrographs at different magnifications. From the obtained micrographs of the CuO–ZnO, the nanocomposite contains several large numbers of densely packed particles without a definite shape, which is caused by agglomeration. On the other hand, the SEM micrograph of CuO nanoparticles in Figure 4b showed that they are mostly composed of uniform nanoflakes that are aggregated in a repetitive pattern, with interconnected thin flakes similar to other report [46]. Additionally, the SEM micrograph of ZnO Nps in Figure 4c shows densely stacked short hexagonal rod-like structures that are randomly distributed similar to earlier report [47,48]. Furthermore, from the obtained TEM images in Figure 5c, CuO–ZnO shows an array of spherical-shaped particles with a diameter ranging from 20–32 nm, which are somewhat arranged like a short nanorod. The morphological properties observed in the CuO–ZnO nanocomposite are indicative of the individual properties of the respective metal oxide constituent as evident in their TEM images in Figure 5. The morphology of the ZnO and CuO nanoparticles was found to be rod-like and spherical, respectively. However, both are found enmeshed in a flat matrix, which also brought about some agglomeration [49], perhaps due to the high temperature of calcination. The particle size of CuO nanoparticles obtained from the TEM micrograph was in the range of 30–50 nm [46].

**Figure 6:**
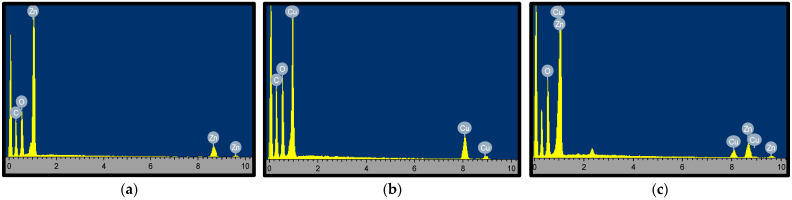
EDX spectrum (**a**) CuO (**b**) ZnO (**c**) CuO–ZnO showing the weight % of elemental constituents in the nanomaterial.

To further understand the morphological composition of these materials and to ascertain their purity, the EDX analysis of CuO, ZnO, and CuO–ZnO NPs was carried out and the obtained spectra are presented in Figure 6a–c. The EDX spectra for CuO–ZnO showed the presence of copper, oxygen, zinc, and carbon. Likewise, in ZnO and CuO NPs, the respective elemental constituents of each nanoparticle were found. The observed C peak in the EDX spectra could be ascribed to the used carbon tape for holding the sample [50]. Additionally, the observed oxygen peak found further confirms that copper and zinc are found in their oxidized form [44]. Moreover, no impurity peak was found in all the spectra which is suggestive of the purity of the prepared nanomaterials. The weight percentage therefore for the elemental constituent of the nanoparticles and their composite is summarized in Table 1.

### 2.4. Biological Studies

#### 2.4.1. Antioxidant Study

**Figure 7:**
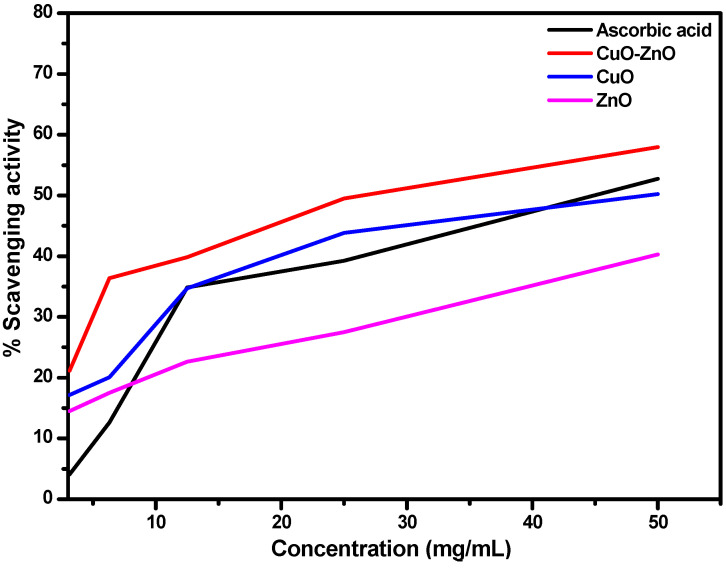
Antioxidant activity of CuO, ZnO and CuO–ZnO nanocomposite prepared using Kei apple leave extract.

Free radicals are formed in many biological systems, and one of the fundamental studies and applications of nanoparticles is their usage in the removal of these radicals, which emanates from the interaction of biomolecules with molecular oxygen [51]. Different methods have been used for this assay, but a notable method often used to ascertain the scavenging potential of several synthetic and natural compounds is the use of the 2,2-diphenyl-1picrythydrazyl hydrate (DPPH) method. This free radical scavenging method involves the use of DPPH, which is a standard nitrogen-concentrated free radical often for the relaxation of the intensive scavenger function in many compounds. The relaxation causes a reduction in the intensity of the DPPH, through the hydrogen/electron transfer acceptance [51]. In this study, the antioxidant activity of CuO, ZnO NPs, and CuO–ZnO nanocomposite was determined by using a DPPH assay, and the percentage of the radical activity was measured at 515 nm and is presented in Figure 7 and Table 2. These results were compared to the standard ascorbic acid. It could be noted from the results presented in Table 1, that as the concentration of the prepared NPs increases, the scavenging efficiency also increases including the standard ascorbic acid [52,53]. This thus implies that the best scavenging concentration in all the samples was 50 mg/mL, but CuO–ZnO NPs showed a 57% scavenging potential which is approximately 5% more than ascorbic acid, 8% more than CuO, and 17% more than ZnO. Furthermore, at 25 mg/mL, CuO also gave scavenging activity of 44% which is better than ascorbic acid. Nevertheless, from the collected data summarized in Table 2 and Figure 6, the CuO–ZnO nanocomposite showed a better scavenging potential (with the minimum inhibitory concentration (IC_50_) values of 3.92 mg/mL) than the individual metal oxide nanoparticles of CuO NPs (6.76 mg/mL), ZnO NPs (8.99 mg/mL) and ascorbic acid (4.96 mg/mL). This showed that the as-prepared CuO–ZnO nanocomposite has better antioxidant properties than the individual metal oxide and the standard ascorbic acid. The enhanced scavenging potential of the as-prepared CuO–ZnO nanocomposite may have emerged due to the increased surface-to-volume ratio and small particle size estimated for the CuO–ZnO nanocomposite in the TEM and XRD analysis [52]. Furthermore, the synergistic antiradical activities of both CuO and ZnO nanoparticles as shown in the estimated IC_50_ of the respective metal oxide could be another reason for the enhanced activity in the nanocomposite. Although, several studies have reported the synthesis and the usefulness of CuO–ZnO nanocomposite, only a few have attempted to study their antioxidant potentials. An example is the reported nanoparticles from Tulsi extract (*Ocimum tenuiflorum*), Neem extract (*Azadirachta indica*), and Aloe Vera extract (*Aloe barbadansis*), that showed that the activity profile was concentration-dependent, which was similar to the results observed in our current study [52]. This report concluded that the composite from the Aloe extract showed the best antiradical activity [52]. Table 3 presents a summary of different scavenging properties on the individual metal oxide nanoparticles. Our current study, therefore, augments the existing information on the antioxidant efficacy of these nanoparticles and further confirms that CuO–ZnO nanocomposite can be a leading scavenging agent upon further clinical trials.

#### 2.4.2. In Vitro Cytotoxicity Studies Using MTT Assay

**Figure 8:**
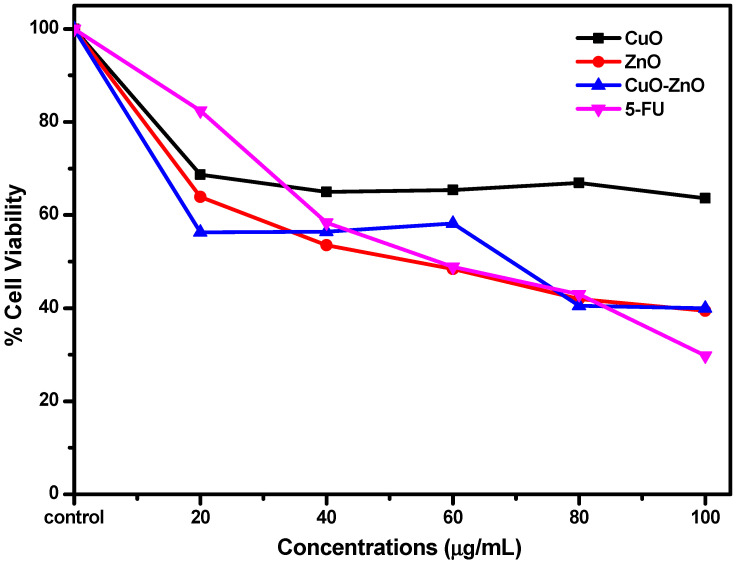
The plot of percentage (%) cell viability of MCF7 breast cancer cell line against concentration for CuO, ZnO, and CuO–ZnO NPs.

The cytotoxic efficacy of trace elements against cancerous cells has made metals and metal oxides NPs potential anticancer agents in the fight against this menace [32]. Examples of these metals include Cu, Zn, Fe, Cr, Mn, I, Se, Cu, and Mo which have all been found in the structure of proteins, DNA, metalloenzymes, hormones, and antioxidant functions. Interestingly, the variation of both Cu and Zn levels in the body was reported to play a vital role in the diagnosis and treatment of cancer [32]. To this end, the potentials of the nanoparticles (CuO and ZnO) and the CuO–ZnO nanocomposite as cytotoxic agents were evaluated against the MCF7 breast cancer cell line, and the obtained activities, presented in Figure 8, were compared to the standard drug, 5-Fluorouracil. Similar to our previous reports and as shown in Figure 8, a concentration-dependent profile was observed and the cytotoxic activity of the CuO–ZnO (3.87 µg/mL) nanocomposite was found to be comparable to the used standard drug (3.71 µg/mL) as shown in the estimated IC_50_ values obtained. The obtained IC_50_ for the CuO and ZnO NPs are 4.04 and 3.90 µg/mL, respectively. The observed result showed that the CuO–ZnO nanocomposite showed the best potential compared to both individual metal oxide components which may be due to the synergy created from the different modes of action exhibited by both CuO and ZnO nanoparticles. ZnO NPs were reported to possess the ability to inhibit cancerous and bacteria cells, by inducing intracellular ROS generation and activating apoptotic signaling pathways, which in turn makes ZnO NPs a good candidate as an anticancer agent [58]. Furthermore, their ability to promote bio-availability by acting as drug carriers can help to adequately enhance therapeutic efficiency [58]. Similarly, other reports have suggested that CuO NPs could induce apoptosis through ROS generation in cancerous cells by modulating the P53 and Bax/Bcl-2 uptake, as in the case of the study involving K562 cells [33]. As already suggested in other studies, the possible mechanism of action follows the destruction of ROS generated during the proliferation of cancerous cells, which are transported as radicals or electrons [43].

## 3. Materials and Methods

### 3.1. Collection and Preparation of Plant Materials

Kei apple leaves were collected fresh from the thorny stalks at the North-West University Garden, Mafikeng, and were correctly identified to be *Dovyalis caffra* leaves. They were washed thoroughly with tap water, followed by double distilled water to remove all unwanted substances. This was followed by drying in the laboratory for three weeks and was grounded into a fine powder. The obtained powder (100 g) was then mixed with deionized water (1 L) in a water bath while maintaining a heating temperature of 80 °C for 2 h. The resulting extract was filtered using a Whatman filter paper with a retention size pore of 11 μm.

### 3.2. Synthesis of CuO Nanoparticle Using Leave Extracts of Dovyalis caffra

The synthesis of the CuO nanoparticles followed a reported procedure with slight modifications [59]. About 50 mL of the extract was mixed with 150 mL of 1 mM of CuSO_4_‧5H_2_O and the pH was raised to an alkaline condition (pH 10). Afterward, the solution was heated to 85 °C and maintained for 1 h while stirring steadily [60]. A color change from yellowish orange to dark green indicated the formation of CuO nanoparticles. The obtained product was then centrifuged for 15 min at 10,000 rpm and the precipitates were washed with deionized water and ethanol. The collected solids were dried in an oven for 12 h at 50 °C and then calcinated for 2 h in a muffle furnace at 400 °C.

### 3.3. Synthesis of CuO Nanoparticle Using Leave Extracts of Dovyalis caffra

The synthesis of ZnO nanoparticles followed a similar procedure as in the case of CuO. About 50 mL of the extract was mixed with 150 mL of 1 mM of Zn(CH_3_CO_2_)_2_·2H₂O and the pH was raised to an alkaline condition (pH 10). Afterward, the solution was heated to 85 °C and maintained for 1 h while stirring steadily [60]. A color change from yellowish orange to a snowy white color indicated the formation of ZnO nanoparticles, which were collected after washing and centrifuging at 10,000 rpm for 15 min. This step was followed by drying in the oven at 50 °C and then calcinated in a furnace for 2 h at 400 °C.

### 3.4. Synthesis of CuO–ZnO Nanoparticle Using Leaf Extracts of Dovyalis caffra

In the preparation of CuO–ZnO nanocomposite with the mole ratio 1:5, about 50 mL of the *Dovyalis caffra* leaf extracts were mixed with 200 mL of Zn(CH_3_CO_2_)_2_·2H₂O (18.35 g, 0.1 mol). This was followed by the adjustment of the pH of the solution to 10 by dropwise addition of an aqueous solution of NaOH. The resulting mixture was vigorously mixed and heated for 30 min until a white solution was observed. Thereafter, the 100 mL solution of CuSO_4_‧5H_2_O (3.19 g, 0.02 mol) was added dropwise to the stirring mixture at 85 °C which then changes to a dark green color. This mixture was allowed to stir for 2 h. This mixture was collected after washing and centrifuging at 10,000 rpm for 15 min, followed by drying in the oven at 50 °C and then calcined in a furnace for 2 h at 400 °C.

### 3.5. Structural and Morphological Studies

The crystalline phase of the prepared nanoparticles was characterized using a Rőntgen PW3040/60 X’Pert Pro XRD diffractometer equipped with nickel filtered Cu Kα radiation (k = 1.5418 Å) at room temperature and a scanning rate of 0.0018° min^−1^. The morphological properties were characterized using a TECNAI G2 (ACI) in both the scanning and transmission electron microscopy with an accelerating voltage of 200 kV. The available functional group in the plant extract, which acted as stabilizing and reducing agents, was studied using Bruker Alpha (FTIR) spectrophotometer. The elemental composition and the morphology of the as-prepared nanomaterials were studied using FEI Quanta FEG 250 field emission gun microscope operating at 15 kV with the Energy dispersive X-ray (EDX) spectra obtained using Oxford Inca software.

### 3.6. Free Radical Scavenging Activity Using DPPH Assay

The 2,2-diphenyl-1-picrylhydrazyl hydrate (DPPH) free radical scavenging capacity of the CuO nanoparticles followed a reported standard procedure [61]. This method was employed to estimate the percentage scavenging capacity of the prepared nanoparticles and their composites. The stock solution of the DPPH was prepared in 100 mL of methanol and kept in the dark for 30 min. Subsequently, 50 mg/mL stock solution of the nanoparticles and their composites was prepared in methanol. This was serially diluted to 25, 12.5, 6.25, and 3.12 mg/mL. About 250 μL of the DPPH mixture was pipetted in triplicates into a 96-well microplate and absorbance was measured at 515 nm against the control containing 1 mL of methanol with the aid of a microplate reader model 680-BIO-RAD, made in the USA. Ascorbic acid was used as a standard drug over the same concentration range. The percentage of the free radical scavenging activity of the NPs was estimated using the equation:

(2) $$\%\;\mathrm{Inhibition}\;\mathrm{of}\;\mathrm{DPPH}\;\mathrm{radical}=\frac{A_{\mathrm{control}}-A_{\mathrm{sample}}\;}{A_{\mathrm{control}}}\times 100$$

where *A*_control_ is the absorbance of the control reactions (containing all reagents except the test compound), while *A*_sample_ is the absorbance of the test compound.

### 3.7. Cytotoxicity Evaluation Using MTT Assay

The in vitro anticancer studies using MCF7 breast cancer cell lines obtained from the ATCC, Manassas, USA, were performed according to a similar reported procedure [59]. The cells were cultured in 25 cm^2^ tissue culture flasks in EMEM which already contained 10% fetal bovine serum 100 µg mL^−1^ penicillin, and 100 μg mL^−1^ streptomycin. The MTT assay in a 96-well plate containing 2.5 × 10^2^ cells/well in 100 μL EMEM cell were used to investigate the viability of the MCF7 cells. Afterward, the prepared cells were incubated overnight at 37 °C. This was followed by the replacement of the medium and subsequently, the addition of samples at various concentrations (20, 40, 80, and 100 μg/mL). These were then incubated for 48 h at 37 °C, followed by the MTT assay. Untreated cells were used as positive control 1 and the untreated cell with DMSO were used as positive control 2, while 5-Fluorouracil was used as standard. A fresh medium containing 10% MTT reagent was used to replace the medium in the assay followed by incubation at 37 °C for 4 h. This was removed, and the insoluble formazan crystals were dissolved in 100 μL of DMSO followed by the absorbance readings taken at 570 nm using DMSO as a blank. The experiment was carried out in triplicate. 

## 4. Conclusions

The desire to synthesize more biocompatible and environmentally friendly nanomaterials has brought about the use of different plant materials for nanoparticle synthesis. Leaf extracts of *Dovyalis caffra*, popularly known as Kei apple, have successfully mediated the synthesis of both CuO and ZnO nanoparticles, and their composite (CuO–ZnO) by acting as a stabilizing agent due to the presence of useful phytochemicals present in their extracts. These crystalline structures and purity of these nanomaterials was confirmed using XRD analysis, which revealed that the obtained diffraction patterns for the three prepared materials were reflection of a monoclinic CuO and hexagonal ZnO phase. The particle sizes of the prepared materials, using an electron microscope, were in agreement with those estimated from the XRD peaks which are found in the nano-scale range. The composite revealed better crystallinity and morphological properties than the respective CuO and ZnO NPs, as shown by the intensity of peaks in the diffractogram, in addition to the observed shape and sizes in the micrograms. The antioxidant and cytotoxicity property studies of these materials were carried out, and both reflected a concentration-dependent pattern in the respective study, with the nanocomposite having a better activity than the individual metal oxides. Although, the CuO–ZnO nanocomposite showed slightly better antioxidant activity than the corresponding metal oxides and the standard ascorbic acid, its cytotoxic properties were slightly less than 5-Fluorouracil. Therefore, CuO–ZnO nanocomposite could be considered as a medicinal agent upon further study owing to their good antioxidant and cytotoxic properties, which may have stemmed from the activity synergy of both CuO and ZnO nanoparticles.

## Figures and Tables

**Table 1:** The percentage weight content of the different elemental compositions of CuO, ZnO, and CuO–ZnO NPs.

Samples	Elemental Constituent of the Nanoparticles in Weight %		
	Zn	Cu	O
CuO	-	72.17	27.83
ZnO	75.03	-	24.97
CuO–ZnO	55.55	17.45	27

**Table 2:** In vitro DPPH assay results at different nanoparticles concentration of the prepared samples and ascorbic acid.

		Concentration in mg/mL					
		3.13	6.3	12.5	25	50	IC_50_
Ascorbic acid	Mean ± SD	0.189 ± 0.02	0.243 ± 0.01	0.261 ± 0.02	0.349 ± 0.01	0.384 ± 0.01	
	% Scavenging activity	4.082	12.625	34.875	39.25	52.75	4.96
CuO–ZnO	Mean ± SD	0.523 ± 0.02	0.422 ± 0.01	0.399 ± 0.01	0.335 ± 0.03	0.279 ± 0.03	
	% Scavenging activity	21.16	36.38	39.85	49.50	57.94	3.92
CuO	Mean ± SD	0.397 ± 0.04	0.447 ± 0.02	0.520 ± 0.05	0.637 ± 0.03	0.661 ± 0.05	
	% Scavenging activity	17.15	20.083	34.728	43.849	50.209	6.76
ZnO	Mean ± SD	0.567 ± 0.02	0.647 ± 0.03	0.513 ± 0.01	0.481 ± 0.03	0.396 ± 0.02	
	% Scavenging activity	14.52	17.54	22.66	27.49	40.30	8.99

**Table 3:** Other in vitro DPPH assay studies showing the estimated IC_50_ values of the scavenging activity using different plant extracts.

NPs	Used Plants	IC_50_ Values in µg/mL	Ref.
CuO	*Galeopsidis herba*	4.12 µg/mL	[20]
	*Abutilon indicum* (leaf extract)	40 ± 0.23 µg/mL	[16]
	*Solanum nigrum* (leaf extract)	131.54 µg/mL	[51]
	*Malus domestica* leaf extract	45.90 µg/mL	[54]
ZnO	*Coptidis rhizoma*	No IC_50_ estimation	[55]
	*Aquilegia pubiflora*	178.45 ± 2.64 µg/mL	[56]
	*Cassia fistula*	2853 µg/mL	[57]

## Data Availability

Not applicable.

## Sample Availability

Samples are available from the authors.

## References

[1] Ferlay J., Colombet M., Soerjomataram I., Parkin D.M., Piñeros M., Znaor A., Bray F. (2021). Cancer Statistics for the Year 2020: An Overview. Int. J. Cancer.

[2] Kalia V.C., Patel S.K.S., Cho B.-K., Wood T.K., Lee J.-K. Emerging Applications of Bacteria as Antitumor Agents. Semin. Cancer Biol..

[3] Thun M.J., DeLancey J.O., Center M.M., Jemal A., Ward E.M. (2010). The Global Burden of Cancer: Priorities for Prevention. Carcinogenesis.

[4] Arruebo M., Vilaboa N., Sáez-Gutierrez B., Lambea J., Tres A., Valladares M., González-Fernández Á. (2011). Assessment of the Evolution of Cancer Treatment Therapies. Cancers.

[5] Rossi F., Noren H., Jove R., Beljanski V., Grinnemo K.H. (2020). Differences and Similarities between Cancer and Somatic Stem Cells: Therapeutic Implications. Stem Cell Res. Ther..

[6] Sutradhar K.B., Amin M.L. (2014). Nanotechnology in Cancer Drug Delivery and Selective Targeting. ISRN Nanotechnol..

[7] Zulkipli I.N., David S.R., Rajabalaya R., Idris A. (2015). Medicinal Plants: A Potential Source of Compounds for Targeting Cell Division. Drug Target Insights.

[8] Cox P.A., King S. (2013). Bioprospecting. Encyclopedia of Biodiversity.

[9] Veeresham C. (2012). Natural Products Derived from Plants as a Source of Drugs. J. Adv. Pharm. Technol. Res..

[10] Otari S.V., Pawar S.H., Patel S.K.S., Singh R.K., Kim S.Y., Lee J.H., Zhang L., Lee J.K. (2017). Canna Edulis Leaf Extract-Mediated Preparation of Stabilized Silver Nanoparticles: Characterization, Antimicrobial Activity, and Toxicity Studies. J. Microbiol. Biotechnol..

[11] Ashraf J.M., Ansari M.A., Fatma S., Abdullah S.M.S., Iqbal J., Madkhali A., Hamali A.H., Ahmad S., Jerah A., Echeverria V. (2018). Inhibiting Effect of Zinc Oxide Nanoparticles on Advanced Glycation Products and Oxidative Modifications: A Potential Tool to Counteract Oxidative Stress in Neurodegenerative Diseases. Mol. Neurobiol..

[12] Agarwal H., Nakara A., Shanmugam V.K. (2019). Anti-Inflammatory Mechanism of Various Metal and Metal Oxide Nanoparticles Synthesized Using Plant Extracts: A Review. Biomed. Pharmacother..

[13] Doan Thi T.U., Nguyen T.T., Thi Y.D., Ta Thi K.H., Phan B.T., Pham K.N. (2020). Green Synthesis of ZnO Nanoparticles Using Orange Fruit Peel Extract for Antibacterial Activities. RSC Adv..

[14] Drummer S., Madzimbamuto T., Chowdhury M. (2021). Green Synthesis of Transition-Metal Nanoparticles and Their Oxides: A Review. Materials.

[15] Lu W., Yao J., Zhu X., Qi Y. (2021). Nanomedicines: Redefining Traditional Medicine. Biomed. Pharmacother..

[16] Ijaz F., Shahid S., Khan S.A., Ahmad W., Zaman S. (2017). Green Synthesis of Copper Oxide Nanoparticles Using Abutilon Indicum Leaf Extract: Antimicrobial, Antioxidant and Photocatalytic Dye Degradation Activities. Trop. J. Pharm. Res..

[17] Mohammadi R., Aziz A., Yangjeh H., Bayrami A., Latifi S., Asadollah N. (2018). Green Synthesis of ZnO and ZnO / CuO Nanocomposites in Mentha Longifolia Leaf Extract: Characterization and Their Application as Anti-Bacterial Agents. J. Mater. Sci. Mater. Electron..

[18] Roy S., Rhim J.W. (2019). Carrageenan-Based Antimicrobial Bionanocomposite Films Incorporated with ZnO Nanoparticles Stabilized by Melanin. Food Hydrocoll..

[19] Gholami P., Dinpazhoh L., Khataee A., Orooji Y. (2019). Sonocatalytic Activity of Biochar-Supported ZnO Nanorods in Degradation of Gemifloxacin: Synergy Study, Effect of Parameters and Phytotoxicity Evaluation. Ultrason. Sonochem..

[20] Dobrucka R. (2018). Antioxidant and Catalytic Activity of Biosynthesized CuO Nanoparticles Using Extract of Galeopsidis Herba. J. Inorg. Organomet. Polym. Mater..

[21] Chang Y.N., Zhang M., Xia L., Zhang J., Xing G. (2012). The Toxic Effects and Mechanisms of CuO and ZnO Nanoparticles. Materials.

[22] Mandal A. (2017). Copper Nanomaterials as Drug Delivery System against Infectious Agents and Cancerous Cells. J. Appl. Life Sci. Int..

[23] Ilves M., Kinaret P.A.S., Ndika J., Karisola P., Marwah V., Fortino V., Fedutik Y., Correia M., Ehrlich N., Loeschner K. (2019). Surface PEGylation Suppresses Pulmonary Effects of CuO in Allergen-Induced Lung Inflammation. Part. Fibre Toxicol..

[24] Raizada P., Sudhaik A., Patial S., Hasija V., Parwaz Khan A.A., Singh P., Gautam S., Kaur M., Nguyen V.-H. (2020). Engineering Nanostructures of CuO-Based Photocatalysts for Water Treatment: Current Progress and Future Challenges. Arab. J. Chem..

[25] Khataee A., Kalderis D., Gholami P., Fazli A., Moschogiannaki M., Binas V., Lykaki M., Konsolakis M. (2019). Cu2O-CuO@biochar Composite: Synthesis, Characterization and Its Efficient Photocatalytic Performance. Appl. Surf. Sci..

[26] Asemani M., Anarjan N. (2019). Self-Dual Leonard Pairs Green Synthesis of Copper Oxide Nanoparticles Extract Assessment and Biological Properties. Green Process Synth.

[27] Siddiqi K.S., ur Rahman A., Tajuddin, Husen A. (2018). Properties of Zinc Oxide Nanoparticles and Their Activity Against Microbes. Nanoscale Res. Lett..

[28] Gunalan S., Sivaraj R., Venckatesh R. (2012). Aloe Barbadensis Miller Mediated Green Synthesis of Mono-Disperse Copper Oxide Nanoparticles: Optical Properties. Spectrochim. Acta Part A Mol. Biomol. Spectrosc..

[29] Wang H., Xu J.Z., Zhu J.J., Chen H.Y. (2002). Preparation of CuO Nanoparticles by Microwave Irradiation. J. Cryst. Growth.

[30] Rangel W.M., Boca Santa R.A.A., Riella H.G. (2020). A Facile Method for Synthesis of Nanostructured Copper (II) Oxide by Coprecipitation. J. Mater. Res. Technol..

[31] Nasrollahzadeh M., Maham M., Mohammad Sajadi S. (2015). Green Synthesis of CuO Nanoparticles by Aqueous Extract of Gundelia Tournefortii and Evaluation of Their Catalytic Activity for the Synthesis of N-Monosubstituted Ureas and Reduction of 4-Nitrophenol. J. Colloid Interface Sci..

[32] Cao Y., Dhahad H.A., El-Shorbagy M.A., Alijani H.Q., Zakeri M., Heydari A., Bahonar E., Slouf M., Khatami M., Naderifar M. (2021). Green Synthesis of Bimetallic ZnO–CuO Nanoparticles and Their Cytotoxicity Properties. Sci. Rep..

[33] Shafagh M., Rahmani F., Delirezh N. (2015). CuO Nanoparticles Induce Cytotoxicity and Apoptosis in Human K562 Cancer Cell Line via Mitochondrial Pathway, through Reactive Oxygen Species and P53. Iran. J. Basic Med. Sci..

[34] Duan X., Liao Y., Liu T., Yang H., Liu Y., Chen Y., Ullah R., Wu T. (2020). Zinc Oxide Nanoparticles Synthesized from Cardiospermum Halicacabum and Its Anticancer Activity in Human Melanoma Cells (A375) through the Modulation of Apoptosis Pathway. J. Photochem. Photobiol. B Biol..

[35] Das D. (2016). Multicomponent Reactions in Organic Synthesis Using Copper-Based Nanocatalysts. ChemistrySelect.

[36] Hekmati M. (2019). Application of Biosynthesized CuO Nanoparticles Using Rosa Canina Fruit Extract as a Recyclable and Heterogeneous Nanocatalyst for Alkyne/Aldehyde/Amine-A 3 Coupling Reactions. Catal. Lett..

[37] Aremu A.O., Ncama K., Omotayo A.O. (2019). Ethnobotanical Uses, Biological Activities and Chemical Properties of Kei-Apple [Dovyalis Caffra (Hook.f. & Harv.) Sim]: An Indigenous Fruit Tree of Southern Africa. J. Ethnopharmacol..

[38] Osuntokun J., Onwudiwe D.C., Ebenso E.E. (2018). Aqueous Extract of Broccoli Mediated Synthesis of CaO Nanoparticles and Its Application in the Photocatalytic Degradation of Bromocrescol Green. IET Nanobiotechnol..

[39] Chaka B., Osano A.M. (2019). Analysis of Selected Nutrient Levels at Different Growth Stages of Dovyalis Caffra (Kei-Apple ) Fruits. Int. J. Res. Innov. Appl. Sci..

[40] Adeyemi J.O., Elemike E.E., Onwudiwe D.C. (2019). ZnO Nanoparticles Mediated by Aqueous Extracts of *Dovyalis Caffra* Fruits and the Photocatalytic Evaluations. Mater. Res. Express.

[41] Veisi H., Karmakar B., Tamoradi T., Hemmati S., Hekmati M., Hamelian M. (2021). Biosynthesis of CuO Nanoparticles Using Aqueous Extract of Herbal Tea (Stachys Lavandulifolia) Flowers and Evaluation of Its Catalytic Activity. Sci. Rep..

[42] Jamil S., Tariq T., Khan S.R., Ehsan M.A., Rehman A., Janjua M.R.S.A. (2021). Structural Characterization, Synthesis and Application of Zincite Nanoparticles as Fuel Additive. J. Clust. Sci..

[43] Elemike E.E., Onwudiwe D.C., Singh M. (2020). Eco-Friendly Synthesis of Copper Oxide, Zinc Oxide and Copper Oxide–Zinc Oxide Nanocomposites, and Their Anticancer Applications. J. Inorg. Organomet. Polym. Mater..

[44] Wasim M., Khan M.R., Mushtaq M., Naeem A., Han M., Wei Q. (2020). Surface Modification of Bacterial Cellulose by Copper and Zinc Oxide Sputter Coating for UV-Resistance/Antistatic/Antibacterial Characteristics. Coatings.

[45] Padmavathy N., Vijayaraghavan R. (2008). Enhanced Bioactivity of ZnO Nanoparticles-An Antimicrobial Study. Sci. Technol. Adv. Mater..

[46] Buledi J.A., Pato A.H., Kanhar A.H., Solangi A.R., Batool M., Ameen S., Palabiyik I.M. (2021). Heterogeneous Kinetics of CuO Nanoflakes in Simultaneous Decolorization of Eosin Y and Rhodamine B in Aqueous Media. Appl. Nanosci..

[47] Saravanan R., Karthikeyan S., Gupta V.K., Sekaran G., Narayanan V., Stephen A. (2013). Enhanced Photocatalytic Activity of ZnO/CuO Nanocomposite for the Degradation of Textile Dye on Visible Light Illumination. Mater. Sci. Eng. C.

[48] Vo N.L.U., Van Nguyen T.T., Nguyen T., Nguyen P.A., Nguyen V.M., Nguyen N.H., Tran V.L., Phan N.A., Huynh K.P.H. (2020). Antibacterial Shoe Insole-Coated CuO-ZnO Nanocomposite Synthesized by the Sol-Gel Technique. J. Nanomater..

[49] Soomro R.A., Tunesi M.M., Karakus S., Kalwar N. (2017). Highly Sensitive Electrochemical Determination of Captopril Using CuO Modified ITO Electrode: The Effect of in Situ Grown Nanostructures over Signal Sensitivity. RSC Adv..

[50] Mbenga Y., Mthiyane M.N., Botha T.L., Horn S., Pieters R., Wepener V., Onwudiwe D.C. (2022). Nanoarchitectonics of ZnO Nanoparticles Mediated by Extract of Tulbaghia Violacea and Their Cytotoxicity Evaluation. J. Inorg. Organomet. Polym. Mater..

[51] Muthuvel A., Jothibas M., Manoharan C. (2020). Synthesis of Copper Oxide Nanoparticles by Chemical and Biogenic Methods: Photocatalytic Degradation and in Vitro Antioxidant Activity. Nanotechnol. Environ. Eng..

[52] Vibitha B.V., Anitha B., Krishna P.G.A., Tharayil J.N. (2020). Plant Extracts Assisted Synthesis, Characterization and Antioxidant Properties of ZnO: CuO Nanocomposites. AIP Conference Proceedings.

[53] Mittal A.K., Kaler A., Banerjee U.C. (2012). Free Radical Scavenging and Antioxidant Activity of Silver Nanoparticles Synthesized from Flower Extract of Rhododendron Dauricum. Nano Biomed. Eng..

[54] Jadhav M.S., Kulkarni S., Raikar P., Barretto D.A., Vootla S.K., Raikar U.S. (2018). Green Biosynthesis of CuO & Ag–CuO Nanoparticles from Malus Domestica Leaf Extract and Evaluation of Antibacterial, Antioxidant and DNA Cleavage Activities. New J. Chem..

[55] Nagajyothi P.C., Sreekanth T.V.M., Tettey C.O., Jun Y.I., Mook S.H. (2014). Characterization, Antibacterial, Antioxidant, and Cytotoxic Activities of ZnO Nanoparticles Using Coptidis Rhizoma. Bioorg. Med. Chem. Lett..

[56] Jan H., Shah M., Andleeb A., Faisal S., Khattak A., Rizwan M., Drouet S., Hano C., Abbasi B.H. (2021). Plant-Based Synthesis of Zinc Oxide Nanoparticles (ZnO-NPs) Using Aqueous Leaf Extract of Aquilegia Pubiflora: Their Antiproliferative Activity against HepG2 Cells Inducing Reactive Oxygen Species and Other in Vitro Properties. Oxid. Med. Cell. Longev..

[57] Suresh D., Nethravathi P.C., Udayabhanu, Rajanaika H., Nagabhushana H., Sharma S.C. (2015). Green Synthesis of Multifunctional Zinc Oxide (ZnO) Nanoparticles Using Cassia Fistula Plant Extract and Their Photodegradative, Antioxidant and Antibacterial Activities. Mater. Sci. Semicond. Process..

[58] Jiang J., Pi J., Cai J. (2018). The Advancing of Zinc Oxide Nanoparticles for Biomedical Applications. Bioinorg. Chem. Appl..

[59] Adeyemi J.O., Elemike E.E., Onwudiwe D.C., Singh M. (2019). Bio-Inspired Synthesis and Cytotoxic Evaluation of Silver-Gold Bimetallic Nanoparticles Using Kei-Apple (Dovyalis Caffra) Fruits. Inorg. Chem. Commun..

[60] Zayyoun N., Bahmad L., Laânab L., Jaber B. (2016). The Effect of PH on the Synthesis of Stable Cu2O/CuO Nanoparticles by Sol–Gel Method in a Glycolic Medium. Appl. Phys. A Mater. Sci. Process..

[61] Iftikhar M., Zahoor M., Naz S., Nazir N., Batiha G.E.-S., Ullah R., Bari A., Hanif M., Mahmood H.M. (2020). Green Synthesis of Silver Nanoparticles Using Grewia Optiva Leaf Aqueous Extract and Isolated Compounds as Reducing Agent and Their Biological Activities. J. Nanomater..

